# Come together: Data sharing to support one health responses to vector‐borne disease threats

**DOI:** 10.1111/mve.70096

**Published:** 2026-07-28

**Authors:** Francis Windram, Sarah Kelly, Stanislav Modrak, Isabelle Dietrich, Corrado Minetti, Karen L. Mansfield, Frederik Seelig, Adam M. Fisher, Maisie Vollans, Nina Billows, Fahad Ahmed, Marta Shocket, Susana Campino, Scott Tytheridge, Sara L. Gandy, Estela Gonzalez, Jolyon M. Medlock, Matthew Baylis, Grant L. Hughes, David A. Ewing, Bethan V. Purse, Georgia Kirby, Clare Strode, Arran J. Folly, David Starns, Heather Ferguson, Simon Smith‐Mullally, Helen Roberts, Christopher M. Jones, Hannah Rose Vineer, Marion England, Christopher Sanders, Samraat Pawar, William D. Pearse, Steven M. White, Robert T. Jones, Lauren J. Cator

**Affiliations:** ^1^ Department of Life Sciences Imperial College London Ascot Berkshire UK; ^2^ The Pirbright Institute Pirbright Woking UK; ^3^ The Open University Milton Keynes Buckinghamshire UK; ^4^ Animal and Plant Health Agency (APHA) Addlestone Surrey UK; ^5^ Aarhus University Aarhus Denmark; ^6^ University of Liverpool Liverpool Merseyside UK; ^7^ Infectious Disease Modelling Team, Analysis and Intelligence Assessment, Chief Data Officer Group UK Health and Security (UKHSA) London UK; ^8^ London School of Hygiene & Tropical Medicine London UK; ^9^ Ulster University Coleraine Northern Ireland UK; ^10^ Lancaster University Lancaster Lancashire UK; ^11^ University of Glasgow Glasgow Scotland UK; ^12^ UK Health and Security Agency (UKHSA) Salisbury Wiltshire UK; ^13^ Liverpool School of Tropical Medicine Liverpool Merseyside UK; ^14^ Biomathematics and Statistics Scotland Edinburgh Scotland UK; ^15^ UK Centre for Ecology & Hydrology Wallingford Oxfordshire UK; ^16^ Edge Hill University Ormskirk Lancashire UK; ^17^ University of Leeds Leeds West Yorkshire UK; ^18^ Department for Farming and Rural Affairs (Defra) London UK

**Keywords:** data science, informatics, one health, vector‐borne disease

## Abstract

Vector‐borne diseases (VBDs) are an increasing threat to animal and human health worldwide. Due to the complexity of VBD transmission and ecology, informing responses to VBDs can be challenging. A One Health approach provides a powerful framework for addressing these threats, but its effectiveness depends on timely access to and integration of diverse and often fragmented data categories. Here we outline three key community actions: applying global metadata and data standards, depositing data into global repositories and responsible shared data usage, which can enhance VBD data sharing. We highlight how these data‐sharing practices allow for the development of new informatic infrastructure, established by the One Health VBD Hub project, to facilitate analyses and ultimately enhance our ability to provide timely responses to endemic and emerging VBD threats.

## Graphical abstract

- A One Health framework integrating diverse data across human, animal and environmental sectors is essential for effective surveillance and response is vital for responding to vector‐borne disease risks.
- Communities adopting global data standards, depositing data in shared repositories, and responsible data use can significantly improve VBD data integration and accessibility.
- These good data sharing practices enable initiatives such as The Defra‐UKRI One Health VBD Hub to provide informatic infrastructure to support researchers to respond to these threats efficiently and effectively.

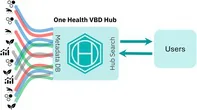

## INTRODUCTION

Vector‐borne diseases (VBDs), which include a variety of bacterial, viral and parasitic infections transmitted mainly by arthropods, are increasing in transmission intensity and global distribution (Gilbert, 2021; Jones et al., 2019; Ryan et al., 2019; Wang et al., 2024). Several high‐profile outbreaks across the world highight the importance of strengthening capacity to detect, monitor, predict and respond to these threats in a timely manner (Cochet et al., 2022; Hung & Huang, 2021; Madder et al., 2011; PAHO, 2024).

VBDs can be particularly challenging to predict and control. In part, this is due to the complexity of their transmission cycles, which can involve multiple vector species and multiple vertebrate hosts. Vertebrates can serve different roles in these cycles: amplifying hosts, which increase pathogen load and can increase the rate of transmission (Haydon et al., 2002); reservoir hosts, which maintain a pathogen over time and can act as a source for transmission into the focal population; maintenance hosts, which are able to support pathogen transmission among themselves or support vector populations in the absence of other species (Gray et al., 2021) and dead‐end hosts, which cannot support onward transmission and act as a transmission sink from the focal population (Hayes, 2019). In addition, the involvement of ectothermic vectors and pathogens with complex life histories and short generations relative to their hosts makes VBD systems highly sensitive to environmental drivers. Changes in variables such as temperature, rainfall and humidity can influence vector and pathogen distribution (Giesen et al., 2023; Ryan et al., 2015; Ryan et al., 2019), phenology (Gilbert, 2021), density (Beck‐Johnson et al., 2013; El Moustaid & Johnson, 2019), competence (Kramer et al., 1983; Paweska et al., 2002) and traits (Pawar et al., 2024).

This complex ecology is further modulated by globalisation and a rapidly changing environmental context. Changes in climate and land use can influence the dynamics of infection through effects on the physiology, behaviour and density of arthropod vectors, pathogens and vertebrate hosts (Plowright et al., 2021). Animal husbandry practices (Métras et al., 2015), wildlife management (Rizzoli et al., 2019) and socio‐economic factors (Halsey et al., 2023; Randolph, 2010) can modify host exposure, susceptibility and competence. In some cases, the presence of disease hotspots, such as regional foci of bovine babesiosis in the United Kingdom (UK) (*Babesia divergens* M'Fadyean & Stockman (Piroplasmida: Babesiidae) transmitted by the widespread tick, *Ixodes ricinus* L. (Ixodida: Ixodidae), is poorly understood despite a relatively detailed understanding of the underlying vector ecology (McFadzean et al., 2023). These cases highlight that multiple factors which contribute to VBD outbreaks need to be considered together to inform risk assessment and response.

A One Health approach requires the ability to integrate data on all contributory factors across multiple systems (Fite et al., 2025; Gittleman, 2024; Johnson et al., 2022). Risk assessments, forecasting and the development of control strategies rely on the community's ability to access and integrate diverse data holdings efficiently. The UK is one example of a place that will increasingly need to address VBD threats. In the past decade, the UK experienced its first detection of West Nile virus (WNV; family: *Flaviviridae*) (Bruce et al., 2025) and first endemic transmission of Usutu virus (USUV; family: *Flaviviridae*) (Folly et al., 2020) and tick‐borne encephalitis (TBEV; family: *Flaviviridae*) (Holding et al., 2019), all of which are vectored by arthropods and all of which are zoonotic with potential to infect both animal reservoir and human populations.

The One Health VBD Hub project (www.vbdhub.org) was co‐funded by the UK Biotechnology and Biological Sciences Research Council (BBSRC) and the Department for Environment, Food, and Rural Affairs (Defra). The project aims to enhance links between data collection, analysis and policy making to inform responses to VBDs (Defra‐UKRI One Health Vector‐Borne Disease Hub, 2023). The Hub supports and leverages best practices in data sharing to support a wide community of VBD practitioners. In the process of developing and establishing the Hub project, we have consulted a wide range of experts in the generation and use of data to inform responses to VBDs. Here, along with members of the UK VBD research and practitioner community, we share insights and discuss steps that all community members can take to improve data sharing, integration and use. These steps and the tools and infrastructure they enable are relevant to the global VBD research landscape and will enhance our ability to respond to these threats under a One Health paradigm.

## CATEGORIES OF VBD DATA

As highlighted above, understanding VBD transmission requires data from a wide range of disciplines across different taxa. The specific data required to inform responses to the emergence or outbreak of a given VBD will vary with transmission biology, current endemic or invasive status, the distribution of susceptible hosts and vectors and many other considerations (Han & Drake, 2016). However, we have found that the *categories* of data that must be integrated are common across VBD systems and can be equally applied to data collected on pathogens, vectors and hosts. The relative importance and subsets of these data most valuable for analysis will also likely vary. As users cannot anticipate every future use case, data are best stored in their raw or most disaggregated form. This allows future users to derive new measures of interest or to reaggregate measures as required for their analysis. Additionally, any given data category requires a minimal subset of metadata to be interpretable and reusable (Table 1).

**TABLE 1:** Examples of global metadata standards and minimum data guidance for key data categories.

Data categories	Metadata standard	Global data repositories	Minimum data guidance
Trait	VecTraits metadata format	VecTraits	MIReVTD (Ryan et al., 2026)
Occurrence	Darwin core	GBIF	MiRead (Rund et al., 2019)
Abundance	VecDyn metadata format	VecDyn	
Genomic	DDBJ/ENA/GenBank feature table	GenBank	MIxS (Yilmaz et al., 2011)
Metagenomic	Sequence Read Archive (SRA) submission guide	Sequence Read Archive	
Transcriptomic	Sequence Read Archive (SRA) submission guide	Sequence Read Archive	
Proteomic	ProteomeXchange consortium guidelines	ProteomeXchange	
Microarray	GEOarchive	Gene Expression Omnibus	
Epidemiological	EpiPulse cases metadata		STROBE (Vandenbrouckel et al., 2007)

*Note*: For major categories of data that are needed for effective vector‐borne disease (VBD) responses, we highlight what we believe to be the most widely accepted and appropriate data standards. We do not attempt to be definitive or list all standards. Species taxonomy (Grenié et al., 2023) and traits (Gallagher et al., 2020) alone, for example, have been the subject of numerous reviews and decades of study.

### Epidemiological data

Epidemiological measures describe the transmission of a pathogen within a defined population and time period of interest. In many cases, this will be humans or domesticated animals, but could also be wildlife or infected vectors. Measures of disease incidence and prevalence are typically reported in terms of the proportion of hosts infected with a particular pathogen in a specified population and time. Many epidemiological measures require detailed information about sampling methods for accurate interpretation.

### Population abundance data

Population abundances are data on the number of individuals. Species occurrences are data on the presence or absence of individuals. Both abundance and occurrence data are typically specific to a location and time point but can be sampled over time and space. These data are often collected for vectors and hosts but may also include free‐living pathogen stages. They can provide vital information about the risk of transmission and associated impact and are often used to understand zoonotic and spillover transmission.

### Trait data

Measurements of phenotypic properties of vectors, pathogens and hosts are classed as trait data. They range from individual‐level traits (e.g. size and metabolic rate), through to interaction traits (e.g. host‐vector, vector‐pathogen and pathogen‐host associations and interaction rate) and population‐level traits (e.g. maximum growth rate). While traits are often defined as characteristics of individuals, here we widen the definition to include the broader properties of species at a population level, as these can be captured with a unified data structure and are sometimes useful for policy‐makers (Gallagher et al., 2020).

### ‘Omic’ data

Underlying many of these measures are ‘omics’ data, including genomic, transcriptomic, proteomic and metabolomic data of vectors, hosts and pathogens. These can be used to predict trait values (phenotypes) and track the occurrence or abundance of species or strains through time and space. Data on population genetics, such as proportions of infections with particular genotypes or the prevalence of resistance alleles at locations in time, can also be captured using the data structures used for ‘omics’. Population genetic estimates can be useful for understanding both the biology and ecology of VBDs, but also for surveillance.

### Environmental data

Environmental data include information on climate, land use, habitat variables and other measures at varying scales. These data are often used to understand and predict how all the above data categories and the associations between them vary across space and time. These can be data on the abiotic and physical aspects of the environment, but can also include information about host or vector populations. For example, socioeconomic data can be mapped and used within broader One Health analyses.

The ability to link these different data categories together has provided key insights to support responses to VBDs (Box 1). Researchers are often required to undertake extensive sampling, experimental work or literature searches to collate the data required to make policy‐relevant projections. When data are collected using public funds, it is often expected and, in many cases, required that these data must be made openly accessible. Individual community members can support data integration by considering how they share the data. Below, we outline three key steps all community members can take to improve our ability to use data to inform responses.

BOX 1 **Integrating diverse data informs responses to VBDs**

Previous One Health collaboration and data sharing in the detections of TBEV and USUV in the UK provide examples of how responses required the simultaneous and joint analysis of multiple data strands.

In 2019, the first occurrence of TBEV was reported in ticks from both East Anglia and Southern England, prior to any confirmed human cases, thanks to proactive surveillance of wildlife and ticks (Holding et al., 2019). Since 2019, up to five probable autochthonous cases in humans had been reported in four separate foci, suggesting TBEV is geographically widespread in British ticks (as reviewed by Callaby et al., 2025; Hassall et al., 2025; United Kingdom Health and Security Agency, 2023a). In the intervening period, the Human Animal Infections and Risk Surveillance (HAIRS) group, a cross‐government committee, performed and proactively updated a qualitative risk assessment on TBEV, as new data emerged. TBE and TBEV data, from surveillance to genomics, have been central to informing the UK Government's response and risk assessments, allowing academia and Government agencies to better understand the likely multiple sources of introductions (United Kingdom Health and Security Agency, 2023a). Since 2022, the notifiable status of acute encephalitis (United Kingdom Health and Security Agency, 2023b), expansion of testing for TBEV in cases of unexplained encephalitis and the addition of TBEV as a differential diagnosis for acute encephalitis (Callaby et al., 2025) have facilitated timely transfer of data between public health agencies and researchers. Here, the ability to link data on tick populations, tick infections, sentinel wildlife exposure, and genetics of the pathogen was integrated to understand drivers and map hotspots of risk to inform targeted response.

Usutu virus was first detected in wild birds at a single site in the UK in 2020 as part of a long‐term surveillance programme (Folly et al., 2020). After this first detection, multiple strands of data were integrated to understand the depth, breadth and impact of the incursion. Data from multiple independent monitoring schemes on vectors and wild birds contributed data on mosquito occurrence and abundance, pathogen prevalence and viral genotype at the index site. These data were combined with reports on diseased bird occurrence from several wildlife monitoring schemes (Garden Wildlife Health, n.d.; National Wildlife Disease Program, n.d.; Predatory Bird Monitoring Scheme, n.d.) and wild bird survival and population data (Breeding Bird Survey, n.d.; Garden BirdWatch research, n.d.) across Great Britain to provide compelling evidence that USUV was circulating in several areas beyond those identified by the national surveillance program, while simultaneously indicating USUV infection was having a significant negative impact on a highly susceptible wild host. These findings, combined with the presence of USUV RNA in surveillance samples, suggested that ongoing virus transmission was likely (Lawson et al., 2022). These data were rapidly integrated into government risk and response assessments, which, in addition to recommending continued surveillance of UK birds and mosquitoes, now advise increased awareness of USUV among clinicians in affected areas and among the public for mosquito bite prevention measures (United Kingdom Health and Security Agency, 2023c).

## APPLY DATA STANDARDS

Data standards define the rules for how data is structured and stored. Some data categories have relatively well‐accepted standards (for example, YYYY‐MM‐DD—year‐month‐day—format for dates in ISO 8601), even if they are not always followed. In addition to the actual data, the way data are described also has standards. Metadata is structured information that enhances the retrieval, use or management of data (National Institutes of Health, 2025). The use of a metadata schema, a system that defines the data elements needed to describe a particular object, enhances the findability and value of data (Contaxis et al., 2022). Fortunately, while in some cases these standards and metadata are still taxon‐specific, many data categories of interest have widely accepted global standards (Table 1). Using standards such as these makes data easier to discover, use and combine to answer questions, increasing our ability to respond to emerging and established VBDs (Pessanha Santos, 2023).

An important issue in VBD data is that some of it may be sensitive and cannot be shared openly; this may be because the data relate directly to animal and human health (UK.gov, 2025). The observation of an infected individual may affect a person's livelihood or one of many data protection and privacy issues. Animal infections can result in significant economic impacts and must also be treated sensitively. There are existing options for sharing these data using anonymisation, which allows sharing while protecting individual identity (Marino et al., 2019; Rodriguez et al., 2022).

However, it is also important to remember that making data discoverable is distinct from making it accessible. Metadata can be shared to make it clear what data are held, with what spatiotemporal resolution and coverage, and to make the process for gaining access transparent without providing access to the data itself (Contaxis et al., 2022). Data catalogues may be used to ensure these data are discoverable. If sensitive data themselves are held in the standardised format, then this will increase how rapidly data can be shared securely when appropriate.

Standardisation improves data consolidation to allow for new insights (Pessanha Santos, 2023). For example, the European Centre for Disease Prevention and Control's VectorNet strongly encourages standardisation in collection protocols and data reporting for abundances and presence or absence of biting midges, ticks, sand flies and mosquitoes (Wint et al., 2023). Its standardised datasets have supported numerous studies, such as modelling the seasonality and timing of peak abundance of *Aedes albopictus* Skuse (Diptera: Culicidae) across Europe (Petrić et al., 2021). OpenDengue, which focuses specifically on dengue, consolidates case data reported by national governments, regional health bodies and international databases and standardises geographic information and reporting structures to allow for easier access and analysis (Clarke et al., 2024). Standardisation reduces the time it takes to implement analyses and new tools on data by ensuring the data are interoperable.

Standardisation can also support the linking of different data categories together by time, space or taxon to gain new insight into VBD systems or forecast VBD dynamics. For example, key insights into the recent detection of USUV in the UK (Lawson et al., 2022) were enabled by the use of common taxonomic, date/time and location metadata, as well as open, shared, and discoverable datasets across domains (trait, abundance and epidemiological) (Box 1). In another example, Brass et al. (2024) integrated mosquito and arbovirus trait and climate data to generate high‐resolution spatial temporal predictions of global dengue risk, extensively validated against mosquito abundance and dengue case data. This depended on being able to link these data together using statistical models to predict how mosquito traits change with the environment, which were then embedded into a mathematical modelling framework driven by climate data to make predictions which were validated against vector abundance data and human case data. These examples highlight the value of standardised data for interpreting and modelling VBD threats in near real‐time. When data standards are not applied consistently, the integration of data becomes more time‐consuming, which prevents them from informing policy at the leading edge of an outbreak. Without discoverable and searchable data, such timely outbreak analysis is challenging or even impossible.

The One Health VBD Hub provides resources and a dedicated curator to support the standardisation of trait, population abundance, presence/absence and epidemiological data. While some of this data has come from recent projects, much of it was previously published but non‐standardised data, which can now be used by the VBD community more easily.

## DEPOSIT DATA IN GLOBAL REPOSITORIES

While publishing data exclusively alongside studies does make it available, it also makes these data hard to find and unify efficiently. Similar issues arise when using taxon‐, region‐ or disease‐specific repositories (Vines et al., 2014). The use of global repositories for data enhances their discoverability (Table 1). Usefully, many of these global repositories use agreed metadata and data standards and offer resources to support users to prepare data for deposition. Thus, using these repositories can also enhance the findability, reuse and interoperability of data.

Data for most species are highly fractured: we know nothing more than the name of 26.7% (c. 93,600) of the 350,699 named plants worldwide (i.e. we have no data on a single location, trait or genetic sample; Cornwell et al., 2019). In our discussions, we have learned that much of the data relevant to VBD responses is similarly fractured (Figure 1). For example, we identified over 40 different locations where data relevant to UK responses might be located. This list is by no means exhaustive, but it gives an idea of the complexity of the current landscape.

**FIGURE 1:**
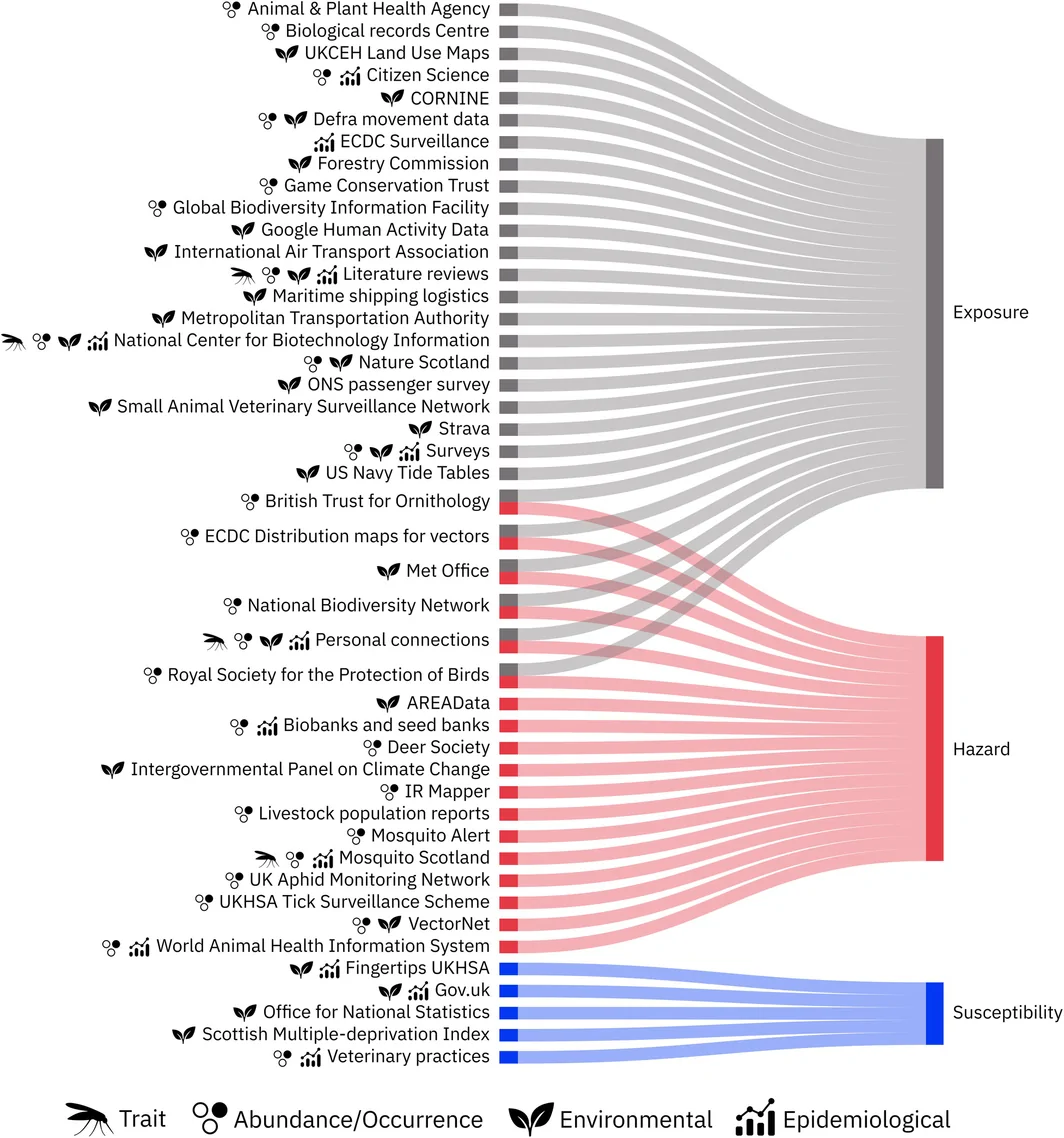
Known data sources identified by UK researchers focused on vector‐borne diseases (VBDs) from research institutes, universities, government and funding agencies. These have been mapped onto different components of VBD risk (Exposure, Hazard and Susceptibility). The icon next to each resource indicates which type of data (epidemiological, trait, abundance/occurrence or environmental). This list is not exhaustive and, given that creators were in the UK, is European‐focused. It does illustrate that even in this group, there are many data sources, many times addressing similar data for different taxa. This lack of consolidation and, in some cases, standardisation, can impede efforts to integrate data among sectors.

While many groups interested in using data are working to bring these fractured data together (GBIF: The Global Biodiversity Information Facility, 2020; Johnson et al., 2023; Rund et al., 2023; Wint et al., 2023), data generators can also contribute by depositing their data in global repositories. A movement toward global repositories will support responses by making it faster to synthesise and identify gaps in data.

The One Health VBD Hub supports users to deposit their data in existing global repositories (Figure 2). For example, trait data has been stored in VecTraits (Johnson et al., 2023), presence/absence data has been deposited into GBIF, and abundance data has been deposited into VecDyn (Rund et al., 2023). This means that data are not only reusable, but they are also easier to discover and access. Further, increasing the representation of the UK in these global resources ensures that these data are considered in global analyses.

**FIGURE 2:**
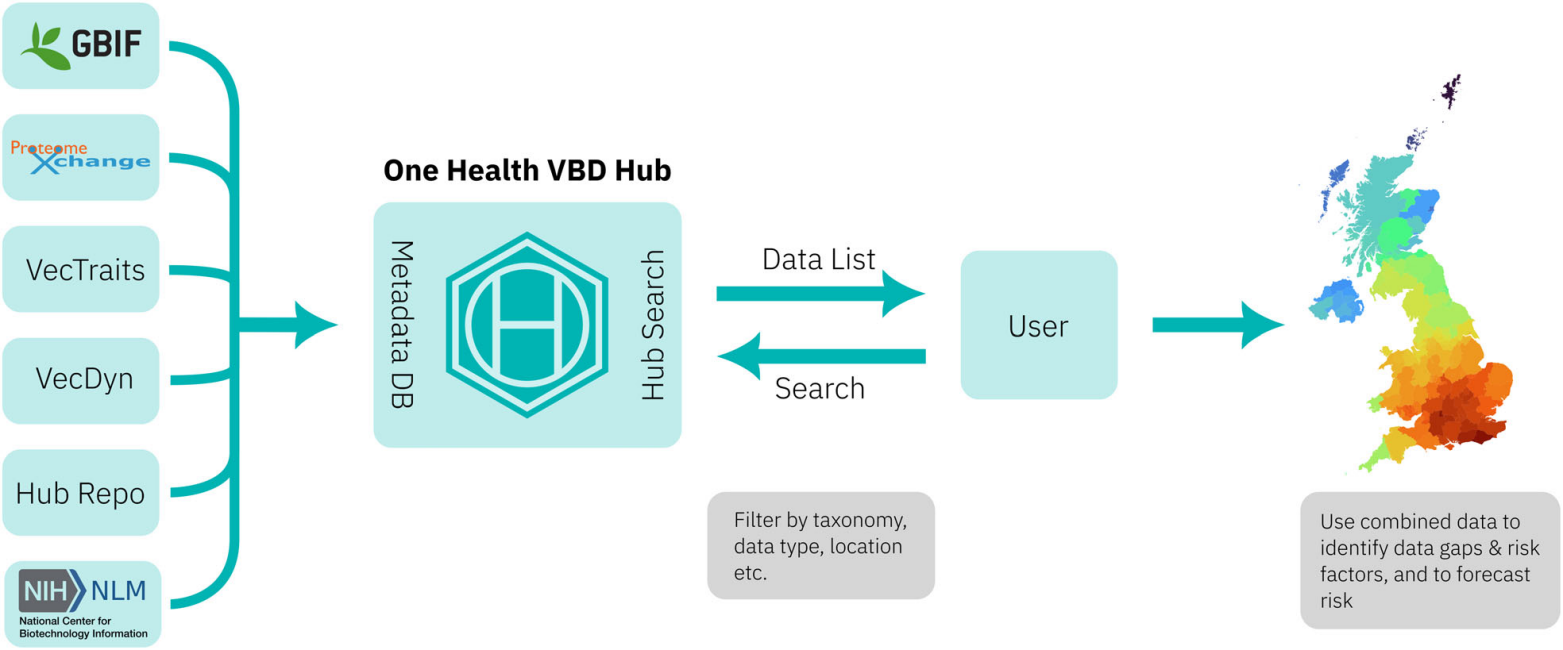
Informatic approach for One Health VBD Hub. Users can use a web interface or programmatic tooling to search for datasets using keywords. The Hub queries the metadata of multiple data repositories through their Application Programming Interfaces (APIs) and retrieves relevant data. The user can filter their searches based on taxonomy, data type and location, informed by the metadata database. The platform also provides tools to support downstream visualisation and integration of datasets over time and space using universal metadata standards (Windram, 2025).

## USE DATA RESPONSIBLY

Data users are as responsible as data depositors for supporting data sharing. In addition to the data protection and sensitivity issues highlighted above, there are important responsibilities for those aiming to re‐use openly accessible data. First, users have a responsibility to use these data appropriately. The onus is on the data user to understand whether the data have been validated and how appropriate it is for use in their analyses. While metadata should be sufficient, in some cases it may not be. Most data standards apply a minimum data standard, which sets the guidelines and formats for reporting a specific category of data (for example, see Rund et al., 2019). While this specifies the minimum information required for data to be reusable, data users may need to access additional information to compare and combine datasets. In these cases, users will need to contact data generators to ensure data are being correctly used.

Second, it is vital that users are transparent about their methods so that all community members understand how data were discovered, accessed and utilised. This includes making code and research software publicly available and applying similar rules to these products as we do to data (Barker et al., 2022). This allows experts to assess the assumptions and robustness of the conclusions drawn.

Finally, data must be appropriately attributed. Two key principles of the Joint Declaration of Data Citation Principles are that (1) data are important, citable outputs of scientific research and (2) citations should be used to give scholarly credit and legal attribution to all contributors to data (Martone, 2014).

To support responsible data use, the One Health VBD project provides openly accessible materials and training (https://vbdhub.org/resources/learn) and hosts community discussions to support the development of a community of practice. It is only when data depositors feel their data will be cited and used properly that they will feel able to share those data.

## DATA AND COMMUNITIES CAN COME TOGETHER

The steps above facilitate what are known as the FAIR principles of data sharing. These are guidelines that make data Findable, Accessible, Interoperable and Reusable (Wilkinson et al., 2016). Sharing data according to FAIR principles allows for the development of technical approaches that increase the value of data. For example, the One Health VBD Hub is developing tools for integrating different data categories to support analyses. The approach takes advantage of metadata standards to allow users to search for trait, occurrence, abundance and epidemiological data from hosts, vectors and pathogens across existing, adopted repositories (Figure 2). These data can then be linked in space, time and taxonomy because they use standardised formats and apply data standards (Figure 2). The platform additionally supports the linking of these data with environmental information at multiple scales (Smith et al., 2022). These tools are only able to work if data are appropriately shared and illustrate the added value of sharing data in this way. While the tools were created in consultation with the UK VBD community, they are applicable to VBD informatics and data analytical challenges globally.

The individual actions of data generators and users paired with the infrastructure such as that provided by the One Health VBD Hub are important parts of the response to emerging VBDs. However, these technical advances are only part of ensuring a robust data ecosystem. Data‐informed policy requires transparent and open communication about data sharing, collection methodology, model limitations and information about interpretation. It is vital that data repositories and tools provide clear, transparent procedures used for decision making. Thus, in addition to building informatic resources, it is vital that we build communities of practice that foster a research culture of transparency, cooperation, collaboration and reproducibility to deliver rapid and agile problem solving. The Hub has prioritised strong governance structures and transparent information for data depositors and users. There are opportunities for positive feedback loops between community and data infrastructure, equity and governance. Strong data management and sharing supports the community in coordinating data collection and gap analyses. Using standards improves data quality and the ability of the community to guide the administration of data access and use.

Given the diversity of data and skills needed to address VBDs, it is unlikely that any one community member will have all required data or expertise to inform responses. As we move forward, we will need to bring together data, models, new ideas and the people who generate them to address these complex challenges. ‘Coming together’ around informatics will increase the impact of data, support interdisciplinary One Health approaches and is essential to timely, effective responses to VBD threats.

## AUTHOR CONTRIBUTIONS

**Francis Windram:** Writing – review and editing; writing – original draft; conceptualization; visualization. **Sarah Kelly:** Conceptualization; writing – original draft; visualization. **Stanislav Modrak:** Conceptualization; writing – original draft; visualization. **Isabelle Dietrich:** Conceptualization; writing – review and editing. **Corrado Minetti:** Conceptualization; writing – review and editing. **Karen L. Mansfield:** Conceptualization; writing – review and editing. **Frederik Seelig:** Conceptualization; writing – review and editing. **Adam M. Fisher:** Conceptualization; writing – review and editing. **Maisie Vollans:** Conceptualization; writing – review and editing. **Nina Billows:** Conceptualization; writing – review and editing. **Fahad Ahmed:** Writing – review and editing; conceptualization. **Marta Shocket:** Conceptualization; writing – review and editing. **Susana Campino:** Conceptualization; writing – review and editing. **Scott Tytheridge:** Writing – review and editing; conceptualization. **Sara L. Gandy:** Conceptualization; writing – review and editing. **Estela Gonzalez:** Writing – review and editing; conceptualization. **Jolyon M. Medlock:** Writing – review and editing; conceptualization. **Matthew Baylis:** Conceptualization; writing – review and editing. **Grant L. Hughes:** Conceptualization; writing – review and editing. **David A. Ewing:** Writing – review and editing; conceptualization. **Bethan V. Purse:** Conceptualization; writing – review and editing. **Georgia Kirby:** Writing – review and editing; conceptualization. **Clare Strode:** Writing – review and editing; conceptualization. **Arran J. Folly:** Conceptualization; writing – review and editing; writing – original draft. **David Starns:** Conceptualization; writing – review and editing. **Heather Ferguson:** Writing – review and editing; conceptualization. **Simon Smith‐Mullally:** Writing – review and editing; conceptualization. **Helen Roberts:** Conceptualization; writing – review and editing. **Christopher M. Jones:** Conceptualization; writing – review and editing. **Hannah Rose Vineer:** Conceptualization; writing – original draft; writing – review and editing; funding acquisition. **Marion England:** Conceptualization; funding acquisition; writing – original draft; writing – review and editing. **Christopher Sanders:** Conceptualization; funding acquisition; writing – original draft; writing – review and editing. **Samraat Pawar:** Conceptualization; funding acquisition; writing – original draft; writing – review and editing. **William D. Pearse:** Conceptualization; funding acquisition; writing – original draft; writing – review and editing. **Steven M. White:** Conceptualization; funding acquisition; writing – original draft; writing – review and editing; visualization. **Robert T. Jones:** Conceptualization; funding acquisition; writing – original draft; writing – review and editing; visualization. **Lauren J. Cator:** Conceptualization; funding acquisition; writing – original draft; writing – review and editing; visualization; supervision; project administration.

## CONFLICT OF INTEREST STATEMENT

The authors declare no conflicts of interest.

## Data Availability

Data sharing not applicable to this article as no datasets were generated or analysed during the current study.
